# Coexistence of large mammals and humans is possible in Europe's anthropogenic landscapes

**DOI:** 10.1016/j.isci.2021.103083

**Published:** 2021-09-03

**Authors:** Benjamin Cretois, John D.C. Linnell, Bram Van Moorter, Petra Kaczensky, Erlend B. Nilsen, Jorge Parada, Jan Ketil Rød

**Affiliations:** 1 Department of Geography, Norwegian University of Science and Technology, 7491 Trondheim, Norway; 2 Norwegian Institute for Nature Research, PO Box 5685, Torgard, 7485 Trondheim, Norway; 3 Department of Forestry and Wildlife Management, Inland Norway University of Applied Sciences, 2480 Koppang, Norway; 4 Department of Mathematical Sciences, Norwegian University of Science and Technology, 7491 Trondheim, Norway

**Keywords:** Earth sciences, Environmental science, Nature conservation

## Abstract

A critical question in the conservation of large mammals in the Anthropocene is to know the extent to which they can tolerate human disturbance. Surprisingly, little quantitative data is available about large-scale effects of human activity and land use on their broad scale distribution in Europe. In this study, we quantify the relative importance of human land use and protected areas as opposed to biophysical constraints on large mammal distribution. We analyze data on large mammal distribution to quantify the relative effect of anthropogenic variables on species' distribution as opposed to biophysical constraints. We finally assess the effect of anthropogenic variables on the size of the species' niche by simulating a scenario where we assumed no anthropogenic pressure on the landscape. Results show that large mammal distribution is primarily constrained by biophysical constraints rather than anthropogenic variables. This finding offers grounds for cautious optimism concerning wildlife conservation in the Anthropocene.

## Highlights

• Biophysical factors had a far greater impact on species distribution than human factors
• This indicates that most species have a broad tolerance of human land use at coarse scales
• We provide grounds for cautious optimism for wildlife conservation in the Anthropocene

## Graphical abstract

**Figure:**
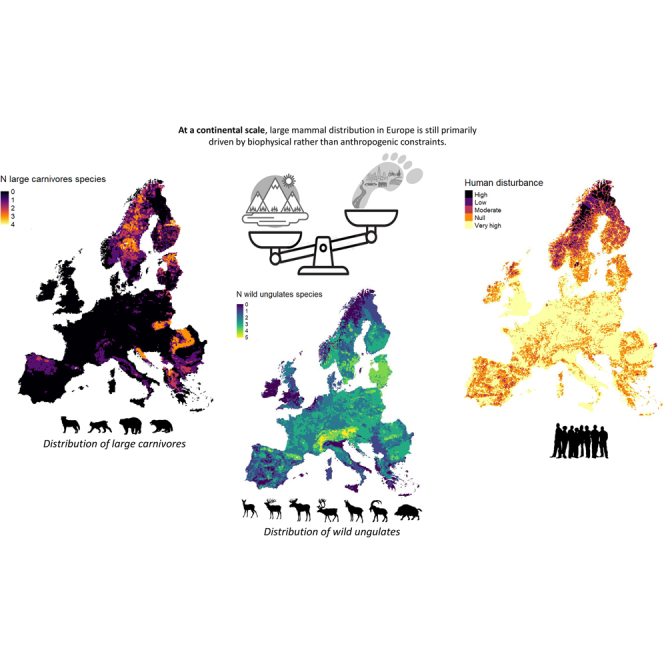

## Introduction

Even though most conservation actions have the primary objective of safeguarding the long-term persistence of wildlife, there is substantial disagreement about the most effective strategies to achieve these goals (e.g., land sparing vs land sharing, Phalan et al., 2011). Some conservationists advocate for implementing a spatial dichotomy, where “wild areas” would be subject to minimal human intervention (land sparing) acting as refugia for wildlife against human disturbance. Another paradigm consists of a diversity of coexistence strategies (land sharing), which envisions the possibility of shared landscapes where human and wildlife interactions are allowed, managed and sustained by effective institutions (Carter and Linnell, 2016; Linnell and Kaltenborn, 2019).

Adopting a land sharing strategy requires a mutual adaptation in behavior from both humans and wildlife (Carter and Linnell, 2016). This may seem especially challenging for large animals as they are more likely to be negatively impacted directly (e.g., through persecution and exploitation) and indirectly (loss and fragmentation of habitats) by human activities owing to their larger spatial and resource requirements and the potential for human-wildlife conflicts (Redpath et al., 2013). Because of their size, large animals with wide-ranging behavior and slow reproductive rates are frequently viewed as being at a disproportionately high risk of extinction (Ripple et al., 2014, 2015).

Coexistence with large mammals has been a historical challenge in Europe. Large carnivores were extensively persecuted in retaliation for killing livestock while large ungulates were overexploited for sport and meat hunting and to minimize damage to crops and forests (Ripple et al., 2014, 2015). This resulted in populations of both taxa being driven to the edge of a near continent-wide extinction in the 19^th^ and early 20^th^ centuries (Chapron et al., 2014; Apollonio et al., 2010). Even though European landscapes are among the most affected by humans (Venter et al., 2016), strict regulations, reintroduction programs, effective wildlife management institutions, reforestation and agricultural abandonment have allowed most large mammal species to recover. Nowadays, these species are again found across very large areas of the European landscape (Chapron et al., 2014; Linnell and Zachos, 2010; Linnell et al., 2020).

Another factor which could potentially have contributed to the re-establishment of these species and their widespread distribution is the widespread protected area network created throughout Europe. However, because of the diverse legislative framework and multiple goals (i.e., encouraging tourism and allowing hunting and different forms of traditional land use) and their small sizes, the conservation effectiveness of protected areas in Europe has been widely disputed for highly mobile, large mammals (Linnell et al., 2015; Gaston et al., 2008).

Although there is an increasing body of literature addressing the influence humans have on large mammals (Tucker et al., 2018; Carter et al., 2012; Alexander et al., 2016), we are not aware of any attempts to quantify the extent to which the contemporary recovering distributions of large predators and their prey in Europe are constrained by the presence of humans' modification in their habitat as opposed to underlying biophysical constraints. The issue is important to understand the factors limiting the potential for large-scale land-sharing in a crowded and human-modified continent.

In this study we evaluate the relative effects of both the human footprint, a proxy for human disturbance levels widely used in large-scale ecological studies (Belote et al., 2020; Tucker et al., 2018, 2021) and protected areas (i.e., to which extent human footprint and protected areas explain species distribution) after accounting for natural heterogeneity. We compare the effect of these two human variables with the effects of biophysical environmental variables such as climate and terrain on large mammal distribution at a continental scale. We use Bayesian hierarchical models to estimate the importance of these variables on species' distributions and compare the environmental niche of these species with and without accounting for human variables by simulating a scenario where the European landscape is free of human influence.

## Results

For ease of interpretation, we consider five disturbance levels (Venter et al., 2016). A ‘no human disturbance’ area has a human footprint of 0; a ‘low disturbance’ area with a human footprint of 1–2; a ‘moderate disturbance’ area a human footprint of 3–5; a ‘high disturbance’ area; a human footprint of 6–11; and ‘very high disturbance’ area with a human footprint of 12–50, following the definition by Venter et al. (2016).

**Figure 1:**
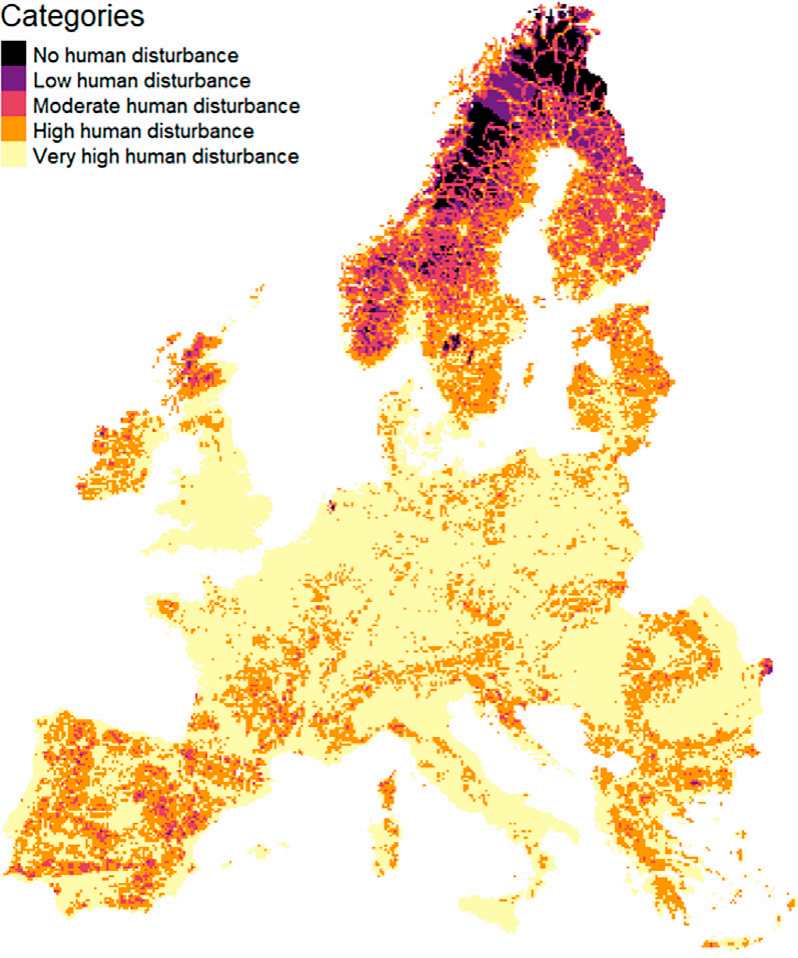
Map of the distribution of human disturbance levels in Europe

With a median human footprint of 12.2, summary statistics show that more than 50% of Europe's area is in an area of very high human disturbance, whereas less than 8% of Europe has no to low human footprints (Figure 1). Protected areas are spread throughout Europe with the median area of protected areas per 100 km^2^ (i.e. per 10 km × 10 km grid cell) being 9 km^2^ (Q1 = 0 km^2^, Q3 = 41 km^2^). Grid cells containing at least 50 km^2^ of protected areas tended to have on average a slightly lower human footprint than grid cells containing less than 50km^2^ of protected areas (median = 10.04 and 12.98 respectively).

**Figure 2:**
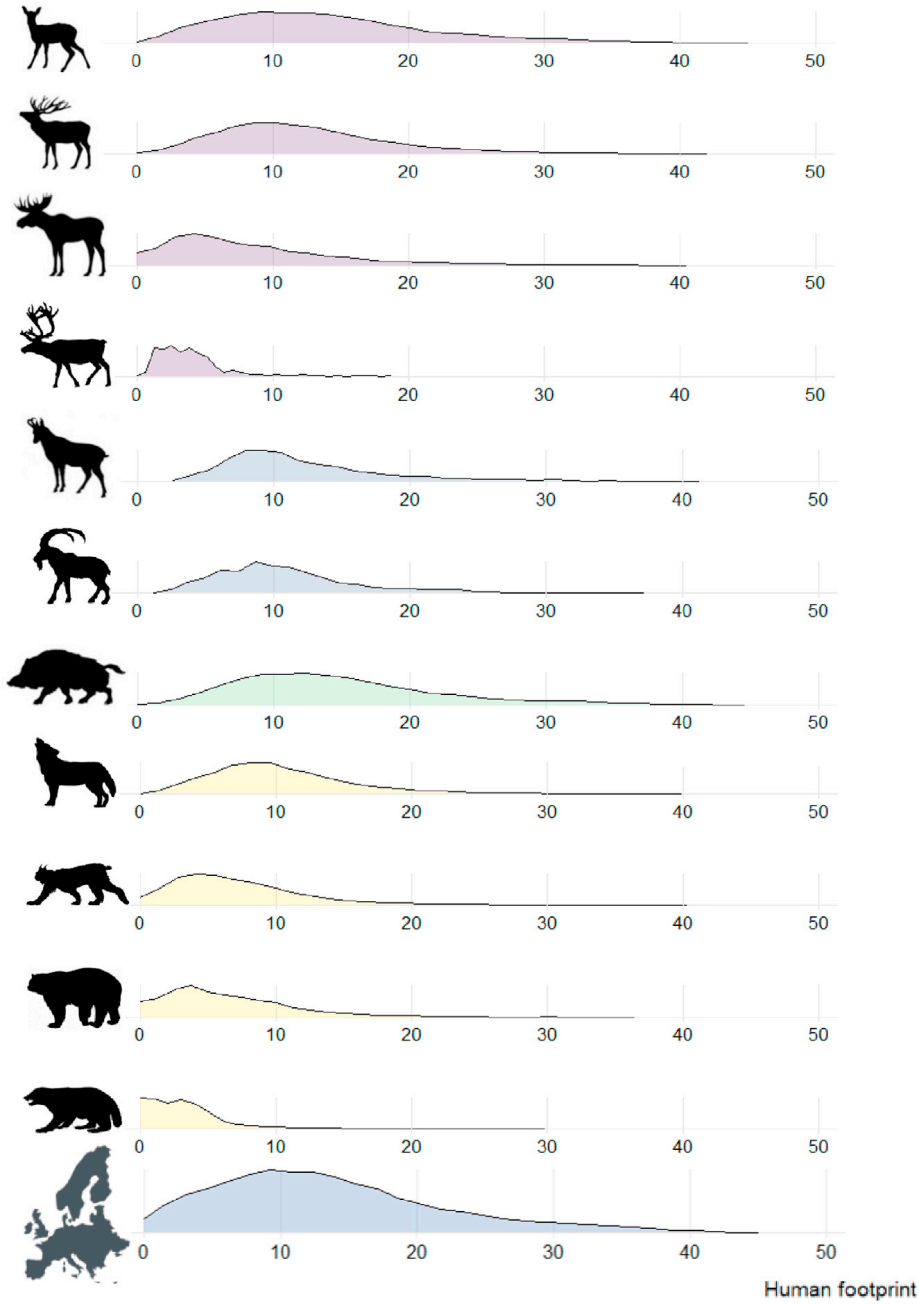
Ridge plot displaying the species' distributions data across the human footprint gradient From top to bottom: roe deer, red deer, moose, wild reindeer, chamois, ibex, wolf, lynx, bear, wolverine, and the European human footprint distribution.

The seven large ungulates and four large carnivores demonstrate great variability in their presence across the human footprint gradient (Figure 2). Roe deer (median of 12.8, Q1 = 8.2, Q3 = 18.2) and wild boar (median = 13.5, Q1 = 9.2, Q3 = 18.7) are the species present at the highest human footprints. These statistics show that more than 50% of the roe deer and wild boar distribution occurs in areas of very high human footprint. Wild reindeer (median = 3.9, Q1 = 2.1, Q3 = 4.8) and wolverines (median = 2.7, Q1 = 1.1, Q3 = 4.4) are at the other end of the spectrum with distributions in places that are least impacted by human disturbance. Our data also shows that wolves are not restricted to “wild” remote places but live in areas where human disturbance is high (median = 9.6, Q1 = 6.8, Q3 = 13). More than 25% of their distribution is in areas where human disturbance is very high.

**Figure 3:**
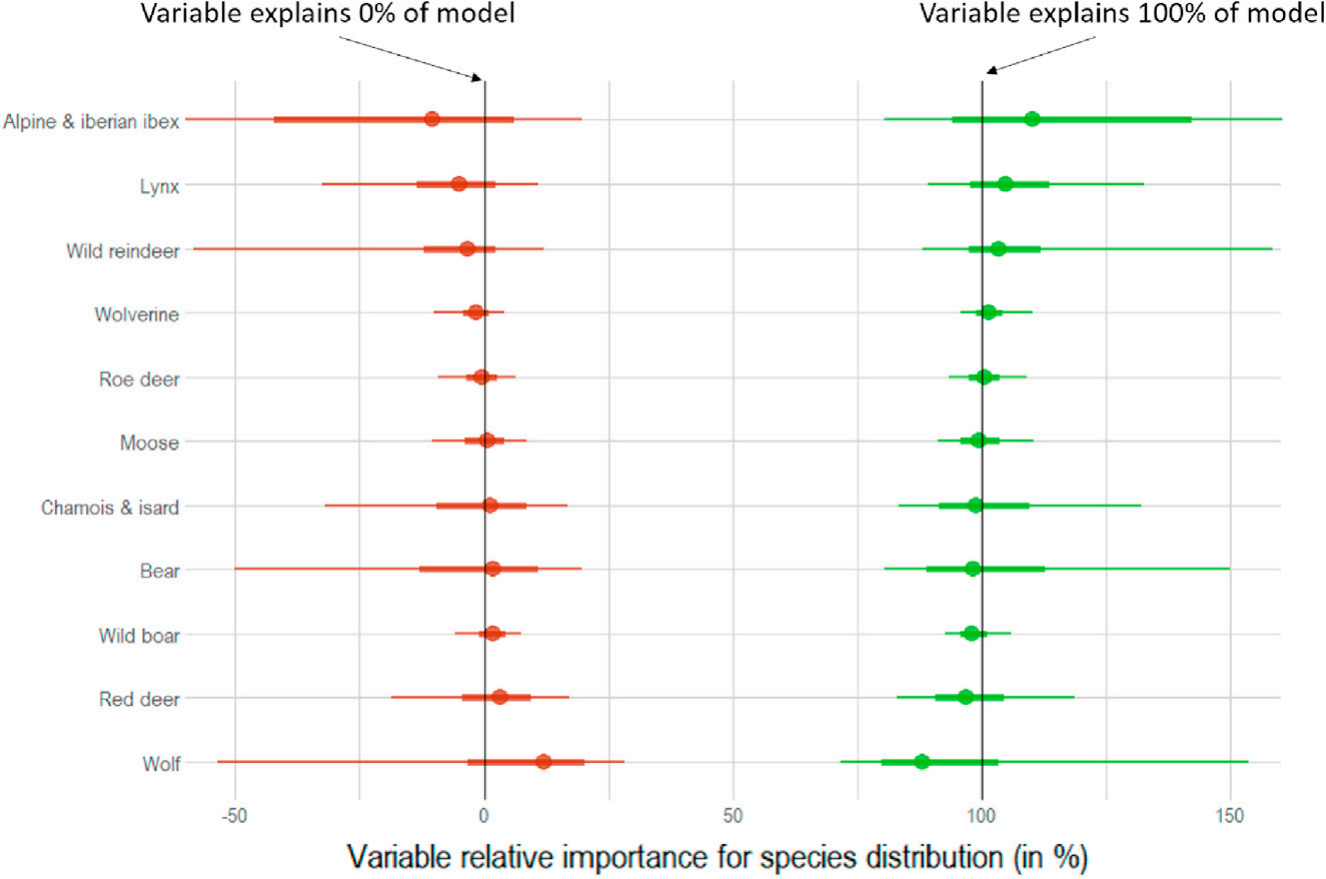
Relative importance for model fit (in percentage) of anthropogenic variables (human footprint and protected area coverage; in red) and biophysical variables (winter and summer severity and terrain ruggedness; in green) to species distribution Negative importance indicates a drop in the R^2^ when the variable is included in the model. Points represent the median value, thick lines represent the 50% credible interval, and thin lines represent the 95% credible interval.

Results from the dominance analysis show that the distributions of all 11 species are largely explained by the biophysical variables (Figure 3). In fact, biophysical variables consistently dominate the models (with a relative importance close to 100%) and the influence of anthropogenic variables in our models is shown to be close to 0% or even negative (i.e., the R^2^ of the model gets worse as we include these variables). Only for red deer and wolf do anthropogenic variables increase the models' R^2^ values (median = 3.3% and median = 12%, respectively), although their effects were still considerably lower than those of the biophysical variables.

**Figure 4:**
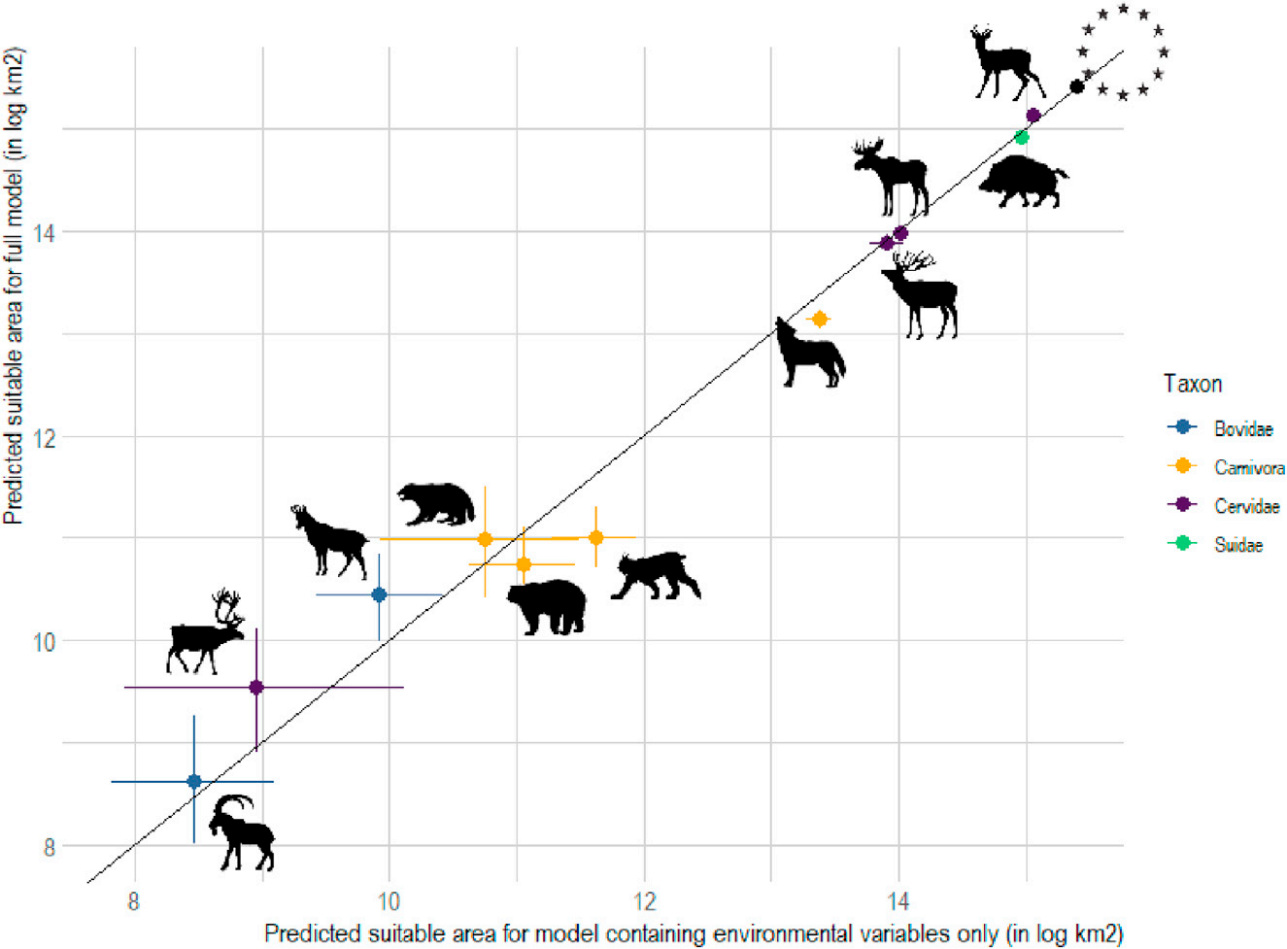
Predicted environmental niche (in log in km^2^) of European large mammals in the presence (y axis) and absence (x axis) of anthropogenic variables A value below the iso-line indicates an increase in potentially suitable area when removing anthropogenic variables. Thin lines represent the 95% credible intervals. The stars represent the area of our study area, Europe. Species symbols, from bottom left, are ibex, wild reindeer, chamois, wolverine, brown bear, Eurasian lynx, wolf, red deer, moose, wild boar, roe deer.

Finally, in Figure 4 we show that human modifications on the landscape hardly influence the area of species' potential distribution. The suitable area for most studied mammals (i.e., ibex, wild reindeer, bears, wolverines, red deer, and moose) is weakly influenced by setting both human footprint and protected areas to zero. Only in the case of chamois and roe deer, we did observe a strong decrease in predicted suitable area when setting the anthropogenic variables to zero (median = −13,900 and −284,400 km^2^, respectively). We also observed a decrease of the predicted suitable area for wolverine, wild reindeer, and ibex when removing anthropogenic effects (median = −12,900 and −6,200 km^2^, respectively), because of the removal of protected areas (see Figures S1 and S2 in the Annexes). In contrast, the total predicted suitable area available for wolf, lynx, and wild boar increases when anthropogenic effects are set to zero (median = 50,700, 133,400 and 131,200 km^2^ respectively). These predicted gains represent 17%, 6%, and 4% of the actual lynx, wolf, and wild boar distributions, respectively.

## Discussion

In this study we have demonstrated that the large-scale distributions of Europe's main large mammalian species include large areas of high to very high human disturbance. Even though there is a wide distribution of high human disturbance combined with a rarity of wild places in the European landscape (Venter et al., 2016) these results show that large mammals can maintain a presence in these heavily modified multi-use landscapes. We have further shown that human disturbance and protected area coverage are only minor drivers of large mammal distributions at the continental scale. Overall, for all large mammals, our results show that the anthropogenic variables are poor predictors of species distribution compared to the other biophysical environmental variables.

Large-scale studies (e.g., with a continental scope) and finer scale studies (e.g., with a sub-national scope) do not answer the same questions, and their results can apparently be in contradiction. Failure to consider scale can lead to misinterpretation of results (Johnson, 1980) and conservation scientists should be careful about the scale used to answer their research questions. Although our models of first order habitat selection (distribution range) suggest that anthropogenic factors such as protected area coverage and human disturbance are minor drivers of large ungulate and large carnivore distribution in Europe, results should not be generalized to higher order habitat selection at finer spatial scales (sensu Johnson 1980). Indeed, many fine scale studies find that the presence or habitat use of large mammals is mainly negatively affected by their proximity to human infrastructure such as trails, roads, or cities (for red and roe deer see D'Amico et al., 2016; Polfus et al., 2011 for moose, Lesmerises et al., 2013 for wolves, Gundersen et al., 2019 for wild reindeer, May et al., 2006 for wolverines, Pęksa and Ciach, 2018 for chamois, Basille et al., 2013 for lynx and Støen et al., 2015 for bear). Furthermore, studies demonstrate that species are often forced to adapt to the proximity of humans through temporal segregation (e.g., animals become primarily night active, Gaynor et al., 2018). As different ecological processes drive distributions at different scales, it is therefore not surprising that results will vary across studies at different scales. For instance, although mountain ungulates forage on steep slopes, human settlements are usually located in the valley bottoms, allowing a vertical coexistence in close proximity. Thus, topographic complexity can provide refuge areas that facilitate human-wildlife proximity (Richard and Côté, 2016). The Human Footprint Index is an aggregated metric of human pressure appropriate for analysis of coarse scale data like ours. Finer scale analyses of other datasets would benefit from breaking down its component layers to explore mechanistic relationships between the different aspects of human activity and land use.

The low effect of anthropogenic variables in our models also implies a weak effect of protected areas on large mammal distributions in Europe. (for ungulates see Linnell et al., 2020, for carnivores Chapron et al., 2014). A main reason is the small size of most European protected areas relative to the spatial requirements of large mammals (for ungulates see Linnell et al., 2020, for carnivores Chapron et al., 2014). Moreover, although European protected areas have on average a lower human footprint, they are not free of human disturbance. In fact, most European protected areas permit harvesting or culling of large herbivores as well as livestock grazing, extensive agriculture, and forestry (van Beeck Calkoen et al., 2020; Linnell et al., 2015), and they encourage tourism. It should be noted that these disturbances are not captured by the Human Footprint Index which focuses on infrastructure, implying that the actual disturbance level of protected areas might be higher than the ones used in this analysis. Only in the case of the wolverine and the wild reindeer does protected area coverage increase the suitable area available because their actual distribution is largely located within protected areas. The mechanistic relationship between the presence of these species and protected area management is however unclear, although for both species human activity and infrastructure has been shown to have negative effects (Nellemann et al., 2000).

This demonstration of the weak effect of human footprint on species distribution compared to the effect of biophysical covariates indicates that most of the large mammals included in our study are flexible enough to adapt to the dramatic anthropogenic impacts which have occurred within their bioclimatic envelope in the European landscape during recent centuries. This is reflected by the overall generalist behavior of these species. For instance, moose seem to adapt to road presence and associated forage in their proximity (Eldegard et al., 2012), whereas agricultural landscapes help roe deer to supplement their diet (Abbas et al., 2011).

### Limitation of the study

Similar to other large-scale studies such as Belote et al. (2020) or Pacifici et al. (2020), our analysis is also limited to distributional data whose quality is highly variable and coarse, and we do not analyze effects on density, behavior or demography. Therefore, while our results document the ability of populations of ungulates and carnivores to persist and use areas in the general proximity to areas of high human footprint, this does not mean these species are not influenced by humans in other ways and at finer spatiotemporal scales. Another challenge is the lack of historical distribution data which makes inferences about causal relationships between human activities and land uses with changes in distributions and population of ungulates populations. Although some attempts to reconstruct large mammals' historical distribution are made, they generally rely on current distribution (Belote et al., 2020)

### Conclusion

Our results contribute to advancing the science of human-wildlife coexistence in the heavily modified landscapes that are typical of the Anthropocene. Although several papers rightly point out that large mammals are threatened by human impacts in many parts of the world (Ripple et al., 2014, 2015) we argue that the European experience demonstrates that coexistence between humans and wild large mammals at broad scales, and continental scale recovery, are both possible. We suggest that it is impossible for nature conservation authorities to rely on a land-sparing policy for large mammals because protected areas large enough to support viable populations of these spaces demanding species don't exist. Ultimately, the challenge of coexistence may not be about whether species are able to cope with human modification to the landscape but whether humans are willing to share their landscape and host wildlife in their backyards (Title and Bemmels, 2018). Europe has multiple layers of formal and informal institutions at continental, national and local scales that effectively manage wildlife and human-wildlife interactions and which appear to have an instrumental role in facilitating this coexistence (Linnell and Kaltenborn, 2019). Overall, the results permit cautious optimism concerning the possibility for wildlife conservation in the Anthropocene.

## STAR★Methods

### Key resources table

**Table:** 

REAGENT or RESOURCE	SOURCE	IDENTIFIER
**Deposited data**		

Wild ungulates distribution data	Linnell et al. 2020	https://doi.org/10.1016/j.biocon.2020.108500
Large carnivores distribution data	Chapron et al. 2014	https://doi.org/10.1126/science.1257553
Terrain Ruggedness Index	Title & Bemmels 2018	https://doi.org/10.1111/ecog.02880
Potential Evapotranspiration	Title & Bemmels 2018	https://doi.org/10.1111/ecog.02880
Snow Cover Duration	Dietz et al. 2015	https://doi.org/10.1080/2150704X.2015.1084551
Human Footprint Index	Venter et al. 2016	https://doi.org/10.1038/ncomms12558
Protected Area	World Database on Protected Areas	https://protectedplanet.net/

**Software and algorithms**		

R Statistical Software	R Core Team, 2020	https://www.r-project.org/
ArcGIS Pro	ESRI	https://www.esri.com/

### Resource availability

#### Lead contact

Further information and requests should be directed to and will be fulfilled by the lead contact, Benjamin Cretois (benjamin.cretois@nina.no).

#### Material availability

This study did not generate new materials.

### Method details

#### Distribution data

In this paper we focus on wild large mammals which are native to Europe and whose distribution is not intensively managed (i.e. doesn't depends on intensive interventions such as the European bison *Bison bonasus,* Linnell et al., 2020). This includes nine large ungulates: roe deer (*Capreolus capreolus*), red deer (*Cervus elaphus*), moose (*Alces alces*), wild reindeer (*Rangifer tarandus*), Alpine chamois (*Rupicapra rupicapra*), Pyrenean chamois (*Rupicapra pyrenaica*), Alpine ibex (*Capra ibex*), Iberian ibex (*Capra pyrenaica*) and wild boar (*Sus scrofa*). We extracted the distribution data provided in Linnell et al. (2020) for all these species. Because the distribution of the mountain ungulates is restricted and because several species belong to the same genus and have similar ecological requirements, we merged the distribution of the Iberian and Alpine ibex, and the distribution of the Alpine and Pyrenean chamois creating *Capra* spp. and *Rupicapra* spp. distributions, respectively. Data come from many sources spread across a period from c. 1990 to 2019. Distribution data for the four species of large carnivore present in Europe; wolves (*Canis lupus*), Eurasian lynx (*Lynx lynx*), brown bears (*Ursus arctos*) and wolverines (*Gulo gulo*) were derived from published data (Chapron et al., 2014), and are derived from the period 2008–2011. Distribution data for all species had a spatial resolution of 10 km × 10 km and take the value 0 if the species is absent and 1 if the species is present. As the underlying distribution data is of widely varying quality and resolution, 10 km × 10 km is the finest resolution we would advocate for large-scale studies as it erases uncertainty related to the location of a species observation and is computationally manageable. In addition, the 10 km × 10 km resolution allows the results of our analysis to be comparable to other large-scale studies such as Tucker et al. (2018) or Chapron et al. (2014). We included data on both herbivore and carnivore distribution from 31 countries, consisting of all EU countries (excluding Cyprus and Malta), plus Norway, Switzerland, Serbia, Albania, Northern Macedonia and the United Kingdom.

#### Explanatory variables

We collected three abiotic covariates, two related to climate and one to terrain relief that are thought to be influential biophysical drivers of species distribution (Araújo and Peterson, 2012). In addition, we included the two anthropogenic covariates human footprint (HF) and protected area. The biophysical drivers represent potential large-scale and long-term constraints on species' potential distributions (i.e. bioclimatic envelopes) operating through physiological tolerance, rather than fine-scaled and temporally variable environmental factors that typically represent vegetation or habitat patch quality.

Terrain Ruggedness Index and the Potential Evapotranspiration for the Warmest Quarter (PETWQ) were acquired from the ENVIREM dataset (Title & Bemmels 2018) at a spatial resolution of 2.5 arc minutes (i.e. about 3 km × 3 km at 50°N). The mean snow cover duration (SCD) was derived from the Global SnowPack, a 14-year average available at a 0.25 km × 0.25 km resolution (from 2000 to 2014, Dietz et al., 2015). We used PETWQ and SCD as proxies for summer and winter severity respectively. Snow cover is widely viewed as being a major limiting factor for species latitudinal and altitude distributions as it correlates with cold winter temperatures, and the physically inhibition of animal movement and access to forage (Leblond et al., 2010). Evapotranspiration serves as a proxy for hot, dry, unproductive summer conditions that also limit species through thermal stress, and poor forage conditions (Tattersall et al., 2012). Terrain ruggedness is widely viewed as being an important escape terrain for species (especially ibex and chamois, and potentially wild reindeer) to avoid disturbance and predation (Nellemann et al., 2007). These three biophysical variables were all obtained as raster data.

As a measure of human disturbance, we chose the Human Footprint Index (HFI version 2009, Venter et al., 2016). Ranging from 0 to 50, the HFI is a composite raster built from multiple variables related to human disturbance (e.g. the extent of built environment, cropland, pasture lands, human population density, nighttime lights, railways, roads and navigable waterways; Venter et al., 2016). The HFI has been recently used in multiple continent-wide comparisons of mammal movement rates (e.g. Tucker et al., 2018, 2021).

Finally, we obtained the protected area coverage from the World Database on Protected Areas: https://protectedplanet.net/). We included all protected areas whose status was listed as either “designated”, “not reported”, “not applicable” or “assigned”. Data was available as vector data and was rasterized at a resolution of 1km^2^ using ArcGIS Pro for ease of computation. We finally used aggregation to sum the total number of 1 km × 1 km pixels of protected area within each 10 km × 10 km grid cell (i.e. the grid cell value for protected area varied from 0 for a grid cell containing no protected area to 100 for a grid cell entirely covered by a protected area). Although European protected areas are almost never wilderness areas (van Beeck Calkoen et al., 2020; Linnell et al., 2015) they are expected to be associated with greater restrictions on human activities that could potentially better limit human impacts on wildlife, and less intensive forms of land use. However, we did not separate the different IUCN categories as previous studies show that there is little difference in human footprint between categories (Leroux et al., 2010) and there is a high degree of variation between European countries in how they manage protected areas of different IUCN categories (Gaston et al., 2008).

We assessed the extent of collinearity between the covariates. Winter and summer severity were negatively related (r = −0.71), as both display strong coastal-inland and north-south gradients. However, we opted to include both as they reflect different mechanisms for species' ecology. Following Dormann et al., 2013 we made sure to carefully interpret the results of these two variables by interpreting the combined effects of all environmental variables (More detailed explanations in Annexes). Other covariates were not significatively correlated with each other (r < 0.70; Table S1 in Annexes). We aggregated all the explanatory variables to the same 10 km × 10 km grid cell resolution.

### Quantification and statistical analysis

#### Model specification

Because the residuals of the non-spatial models were strongly spatially correlated, we fitted an intrinsic conditional autoregression (iCAR) model using hierarchical Bayesian models for each of the 13 species. The probability of presence (*π*) of a given species in a given grid cell was calculated using a Bernoulli distribution and the following model:

$$y_{i}∼Bernouilli\left(\pi_{i}\right)$$

$$logit\left(\pi_{i}\right)=\;\alpha_{i}+\;x_{i}\beta +\;u_{i}$$

where $x_{i}$ is the vector of covariates for cell *i*, β the vector of parameters to be estimated and $u_{i}$ the spatially correlated random effect whose prior is defined as:

$$u_{i}|u_{k}∼\;normal\left(\frac{\sum_{i\neq k}w_{i,k\;}u_{k}}{n_{i}},\;\frac{\sigma_{u}^{2}}{n_{i}}\right)$$

where $w_{i,k\;}=\;$1 if grid cells *i* and *k* are neighbors and 0 otherwise. $n_{i}$ is the total number of neighbors of grid cell *i*. We define two cells as being neighbors if they directly share a single boundary point. All models assume a vague prior for the regression parameters β∼normal(Mean=0,SD=1000) and we used a penalized complexity prior on the spatial effect to avoid risks of overfitting.

As we expect species to have an optimal niche for environmental variables, we included linear and quadratic terms for winter and summer severity and ruggedness (Svenning et al., 2011). We also included linear and quadratic terms for human footprint as we suspected certain species to have an optimal niche in the moderate human disturbance level. We only included a linear effect for protected area coverage as we only expected a linear response.

To fit the spatial models, we used the Integrated Nested Laplace Approximation (INLA) approach with the package R-INLA (Lindgren and Rue, 2015). INLA is a faster alternative to Markov Chain Monte Carlo approaches and yields similar, if not identical, results (Beguin et al., 2012). We standardized the covariates to enable direct comparison between the regression coefficients. All analyses were conducted in R 3.6.1.

We validated the models by plotting residual values against covariates for each model. We also plotted the leave-one out cross validation scores (conditional predictive ordinate CPO in our case) to estimate model fit.

#### Evaluation of variables' importance for species' distribution

We estimated the relative importance of both environmental and anthropogenic variables using dominance analysis (Azen and Budescu, 2003), which is a procedure to quantify the importance of a random variable through examination of the R^2^ values (or similar metrics) for all possible subset models of a predefined full model. In a dominance analysis, the higher the dominance score the more useful is the random variable in predicting the response variable. Because the number of models required to estimate the importance of a single random variable grows exponentially with the total number of random variables, we did not quantify the importance of each single variable, but rather the importance of the combined effect of summer and winter severity and ruggedness (“environmental variables”) and human footprint and protected area coverage (“anthropogenic variables”). Thus, we fitted 3 models for each of the 11 species: a full model containing all variables, a model containing only the environmental variables and a model containing only the anthropogenic variables. For all models we computed the R^2^_glmm_, a modified version of the classic R^2^ which is suitable for mixed models (Nakagawa and Schielzeth, 2013). We sampled 1,000 values from the posterior distribution of the model parameters and bootstrapped the R^2^_glmm_ 1,000 times. We finally rescaled the dominance score for it to range from 0 to 100%.

#### Quantifying the effect of anthropogenic variables on the size of the species' suitable habitat

To further assess the results of the dominance analysis we assessed the relative extent to which anthropogenic variables influence the realized distribution of the studied large mammals we quantified the geographic representation of the suitable habitat for each species (i.e. the potential suitable area available due to environmental predictors only, Guisan and Thuiller, 2005). We predicted the probability of a species' occurrence within a grid cell both when anthropogenic variables were set at their minimum value (i.e. we simulated a landscape free of all human influence: no human footprint and no protected areas) and when anthropogenic variables are set to their observed values. We summed these predicted occurrences across Europe to estimate the expected number of occupied cells (i.e. the size of a species' suitable area in Europe). A sum of predictions in a human-free landscape higher than a sum of prediction for the full model implies that the species increase its range in absence of human influence in the landscape. We sampled 1,000 values from the posterior distribution of the model parameters and bootstrapped the niche area 10,000 times.

## Data Availability

• The dataset and scripts used to conduct all analyses presented in this manuscript are fully available and has been deposited on Open Science Framework (https://doi.org/10.17605/OSF.IO/XV8NH). • Data concerning wild ungulates distribution are fully available and has been extracted from Linnell et al. (2020) (https://doi.org/10.1016/j.biocon.2020.108500). • Data on large carnivores' distribution are fully available and has been extracted from Chapron et al. (2014) (https://doi.org/10.1126/science.1257553).
