# Supplementary material for: Coexistence of large mammals and humans is possible in Europe's anthropogenic landscapes
Source: iScience. 2021 Sep 3;24(9):103083. doi: 10.1016/j.isci.2021.103083 (PMC8455722; doi:10.1016/j.isci.2021.103083)
Supplement: Document S1. Figures S1–S4 and Tables S1–S4 [file mmc1.pdf]

iScience, Volume *24*

Supplemental information

Coexistence of large mammals and humans is possible in Europe's anthropogenic landscapes

Benjamin Cretois, John D.C. Linnell, Bram Van Moorter, Petra Kaczensky, Erlend B. Nilsen, Jorge Parada, and Jan Ketil Rød

Supplementary material for:

Coexistence of dense human population and large mammals is possible in Europe's anthropogenic landscape

This document includes:

Table S1: Pearson correlation coefficients between covariates. Related to the STAR Method.

Table S5: Full models' coefficients. Related to Figure 3.

Table S3: Table of marginal R2. Related to Figure 3.

Table S4: Table of conditional R2. Related to Figure 3.

Figure S1: Environmental niche of European large mammals in presence (y axis) and absence (x axis) of human footprint variables in log km^2. Protected area coverage at normal values. Related to Figure 4.

Figure S2: Environmental niche of European large mammals in presence (y axis) and absence (x axis) of human footprint variables in log km^2. Protected area coverage at maximal value across the entire landscape. Related to Figure 4.

Table S1: Pearson correlation coefficients between covariates. Related to the STAR Methods.

	Winter severity	Summer severity	Terrain ruggedness	Human footprint
Summer severity	-0.71			
Terrain ruggedness	0.14	-0.16		
Human footprint	-0.55	0.3	-0.2	
Protected area coverage	-0.01	-0.02	0.21	-0.15

Table S2: Models coefficients. Related to Figure 3.

Variable	mean	0.025% credible interval	97.5% credible interval
**Bear**			
Intercept	-5.393	-5.901	-4.886
Winter severity	2.344	1.845	2.843
Winter severity^2	-0.529	-0.731	-0.328
Terrain ruggedness	1.096	0.769	1.422
Terrain ruggedness^2	-0.215	-0.309	-0.121
Summer severity	-0.143	-0.662	0.376
Summer severity^2	-0.549	-0.807	-0.29
Human Footprint	-0.393	-0.583	-0.203
Human Footprint^2	-0.031	-0.175	0.112
Protected area	0.03	-0.076	0.137
**Alpine and Pyrenean chamois**			
Intercept	-6.605	-7.109	-6.102
Winter severity	1.728	1.216	2.239
Winter severity^2	-0.765	-1.032	-0.499
Terrain ruggedness	2.03	1.712	2.349
Terrain ruggedness^2	-0.191	-0.275	-0.107
Summer severity	0.319	-0.19	0.828
Summer severity^2	-0.074	-0.329	0.18
Human Footprint	0.32	0.082	0.559
Human Footprint^2	-0.06	-0.183	0.063
Protected area	0.154	0.031	0.278
**Ibex Tot**			
Intercept	-8.7	-9.432	-7.967
Winter severity	0.538	-0.026	1.102
Winter severity^2	-0.047	-0.297	0.203
Terrain ruggedness	1.027	0.726	1.328
Terrain ruggedness^2	-0.033	-0.09	0.025
Summer severity	-0.477	-0.935	-0.021
Summer severity^2	0.045	-0.127	0.217
Human Footprint	0.023	-0.2	0.246
Human Footprint^2	-0.078	-0.215	0.059
Protected area	0.153	0.025	0.282
**Lynx**			
Intercept	-3.307	-3.588	-3.026
Winter severity	3.123	2.806	3.44

Winter severity^2	-0.818	-0.966	-0.67
Terrain ruggedness	0.625	0.367	0.882
Terrain ruggedness^2	-0.219	-0.293	-0.145
Summer severity	-1.028	-1.425	-0.631
Summer severity^2	-0.92	-1.178	-0.661
Human Footprint	-0.505	-0.645	-0.365
Human Footprint^2	0.086	-0.006	0.179
Protected area	-0.124	-0.216	-0.032
**Moose**			
Intercept	-2.219	-2.528	-1.909
Winter severity	6.127	5.807	6.446
Winter severity^2	-2.029	-2.176	-1.882
Terrain ruggedness	-1.264	-1.525	-1.003
Terrain ruggedness^2	0.155	0.077	0.233
Summer severity	-1.493	-1.991	-0.996
Summer severity^2	-0.943	-1.241	-0.645
Human Footprint	-0.009	-0.158	0.139
Human Footprint^2	0.078	-0.005	0.161
Protected area	-0.216	-0.296	-0.135
**Red deer**			
Intercept	-0.305	-0.515	-0.095
Winter severity	1.403	1.194	1.611
Winter severity^2	-1.466	-1.604	-1.328
Terrain ruggedness	0.521	0.343	0.7
Terrain ruggedness^2	-0.088	-0.146	-0.03
Summer severity	0.782	0.628	0.935
Summer severity^2	0.146	0.019	0.273
Human Footprint	-0.161	-0.281	-0.041
Human Footprint^2	-0.008	-0.069	0.052
Protected area	0.271	0.202	0.341
**Roe deer**			
Intercept	5.389	5.106	5.672
Winter severity	6.039	5.753	6.326
Winter severity^2	-2.387	-2.529	-2.244
Terrain ruggedness	-0.291	-0.481	-0.102
Terrain ruggedness^2	0.196	0.123	0.269
Summer severity	3.396	3.241	3.551
Summer severity^2	-0.452	-0.579	-0.325
Human Footprint	0.32	0.18	0.46
Human Footprint^2	-0.033	-0.095	0.03
Protected area	0.223	0.143	0.304

**Wild boar**			
Intercept	2.429	2.229	2.628
Winter severity	1.671	1.483	1.859
Winter severity^2	-1.289	-1.43	-1.148
Terrain ruggedness	0.593	0.428	0.758
Terrain ruggedness^2	0.08	0.033	0.128
Summer severity	4.276	4.128	4.424
Summer severity^2	-0.299	-0.422	-0.176
Human Footprint	-0.315	-0.427	-0.202
Human Footprint^2	-0.012	-0.067	0.043
Protected area	0.316	0.243	0.39
**Wild reindeer**			
Intercept	-15.861	-19.23	-12.495
Winter severity	3.897	2.156	5.638
Winter severity^2	-0.632	-1.065	-0.2
Terrain ruggedness	-0.772	-1.419	-0.127
Terrain ruggedness^2	-0.004	-0.182	0.174
Summer severity	-6.104	-10.994	-1.217
Summer severity^2	-1.555	-3.252	0.14
Human Footprint	-0.747	-1.532	0.036
Human Footprint^2	0.104	-0.402	0.609
Protected area	0.382	0.173	0.591
**Wolverine**			
Intercept	-14.683	-17.772	-11.596
Winter severity	2.481	1.525	3.437
Winter severity^2	-0.378	-0.656	-0.1
Terrain ruggedness	0.669	0.212	1.125
Terrain ruggedness^2	-0.191	-0.322	-0.059
Summer severity	-12.353	-17.121	-7.589
Summer severity^2	-4.414	-6.136	-2.693
Human Footprint	-0.416	-0.993	0.161
Human Footprint^2	0.136	-0.214	0.485
Protected area	0.18	0.04	0.321
**Wolf**			
Intercept	-4.422	-4.882	-3.962
Winter severity	2.072	1.583	2.56
Winter severity^2	-1.04	-1.341	-0.739
Terrain ruggedness	1.328	1.041	1.616
Terrain ruggedness^2	-0.21	-0.287	-0.132
Summer severity	1.048	0.606	1.489
Summer severity^2	-0.459	-0.683	-0.234
Human Footprint	-0.949	-1.124	-0.774

Human Footprint2	0.029	-0.088	0.146
Protected area	0.318	0.217	0.419

Table S3: Table of marginal R^2 for the full models, models containing environmental variables only and models containing anthropogenic variables only. Related to Figure 3.

	Full model	Environmental variables only	Anthropogenic variables only
Wolverine	0.925	0.937	0.069
Wild reindeer	0.887	0.911	0.119
Moose	0.660	0.660	0.190
Wild boar	0.401	0.397	0.165
Lynx	0.326	0.337	0.038
Roe deer	0.263	0.264	0.001
Bear	0.167	0.166	0.034
Alpine & Pyrenean chamois	0.146	0.146	0.030
Alpine & Iberian ibex	0.113	0.123	0.025
Wolf	0.097	0.090	0.046
Red deer	0.048	0.047	0.004

Table S4: Table of conditional R^2 for the full models, models containing environmental variables only and models containing anthropogenic variables only. Related to Figure 3.

	Full model	Environmental variables only	Anthropogenic variables only
Wolverine	0.988	0.990	0.881
Wild reindeer	0.967	0.974	0.784
Moose	0.936	0.937	0.789
Red deer	0.933	0.934	0.936
Lynx	0.921	0.923	0.926
Wild boar	0.918	0.918	0.552
Wolf	0.918	0.922	0.922
Roe deer	0.916	0.914	0.797
Bear	0.905	0.906	0.907
Alpine & Pyrenean chamois	0.858	0.860	0.893
Alpine & Iberian ibex	0.822	0.824	0.846

Figure S1: Environmental niche of European large mammals in presence (y axis) and absence (x axis) of human footprint variables in log km^2. Protected area coverage at current value. Related to Figure 4.

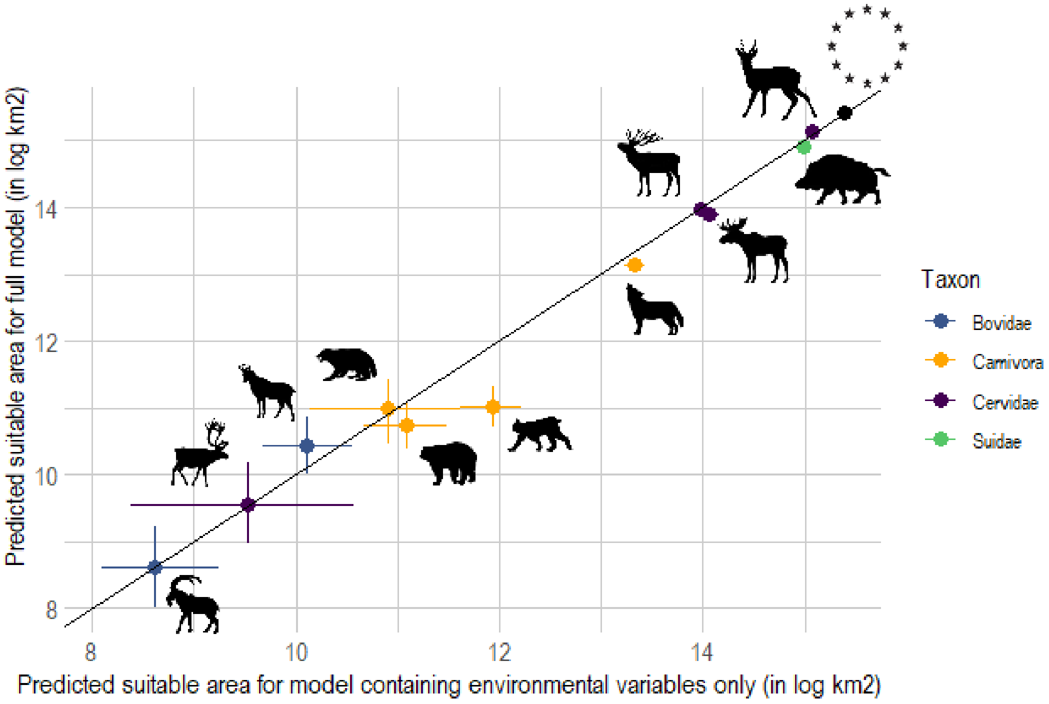

Figure S2: Environmental niche of European large mammals in presence (y axis) and absence (x axis) of human footprint variables in log km^2. Protected area coverage at maximal value across the entire landscape. Related to Figure 4.

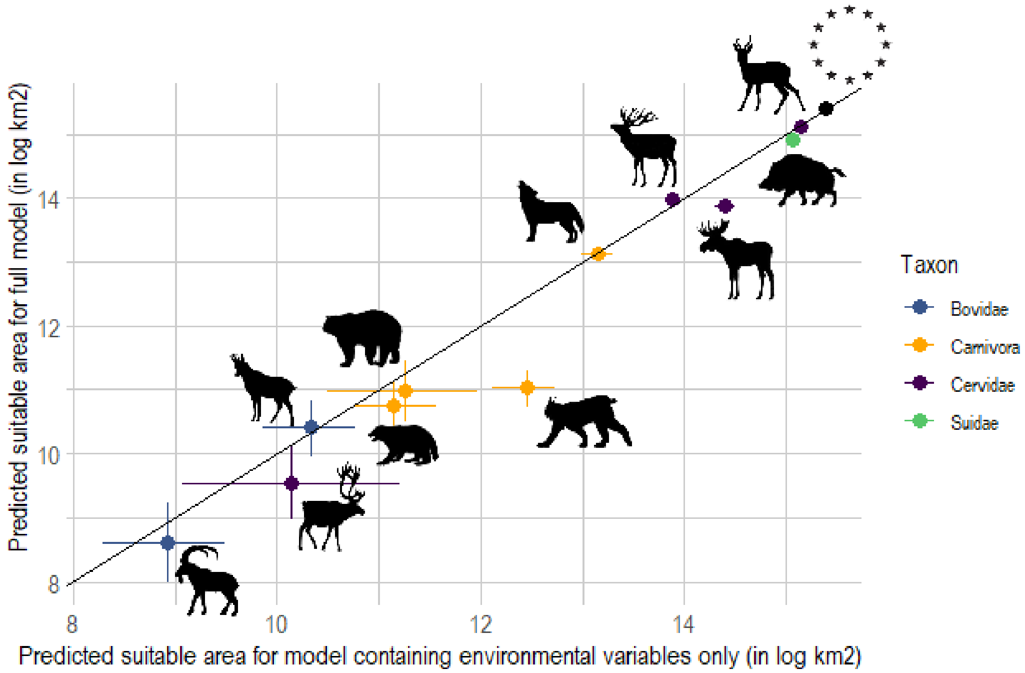
Predicted suitable area for full model (in log km2)
Predicted suitable area for model containing environmental variables only (in log km2)
8
10
12
14
8
10
12
14
Taxon
Bovidae
Carnivora
Cervidae
Suidae
